# The Relationship between Friendship and Social Life of Patients With Type 2 Diabetes with Depression

**DOI:** 10.1192/j.eurpsy.2022.1600

**Published:** 2022-09-01

**Authors:** V. Kourkoumpas, K. Dimou, E. Dragioti, S. Mantzoukas, M. Gouva

**Affiliations:** 1 University of Ioannina, Laboratory Of Psychology Of Patients, Families And Health Professionals)., Ioannina, Greece; 2 University of Ioannina, Nursing, Ioannina, Greece

**Keywords:** Diabetes Melitus, Depression, Patients, Social Life

## Abstract

**Introduction:**

INTRODUCTION: Several studies have shown that the relationship between Diabetes and Depression is significant, but few have evaluated the relationship between this depression and patients’ social life.

**Objectives:**

OBJECTIVE: Exploring the friendships and social life of patients with type 2 diabetes with levels of depression.

**Methods:**

METHODS: The sample consisted of 130 Greek patients with type 2 diabetes and a mean age of 63.28 (SD = 13.89), who completed the following questionnaires voluntarily and anonymously: a) Zung Depression Scale and b) socio-demographic and self-reported questionnaire for their past and present friendships.

**Results:**

RESULTS: Patients who had friends in the past scored lower depression rates (44.63 ± 11.53) than patients who did not have friends in the past (60.50 ± 6.36), with a statistically significant difference between them (p = 0.045), while patients who currently have friends scored lower depression rates (42.91 ± 10.86) than patients who do not currently have friends (58.81 ± 6.07), with a statistically very significant difference between them ( p = 0.000). Patients with type 2 diabetes who are not currently friends have higher levels of depression by 3.8 points compared to patients with type 2 diabetes who are currently friends.

**Conclusions:**

CONCLUSIONS: Patients with diabetes mellitus with low levels of social life show statistically higher rates of depression and further study of this relationship is considered necessary.

**Disclosure:**

No significant relationships.

